# Optimization of Selenium Fortification and Ultrasound-Assisted Extraction of Bioactive Selenium-Polysaccharide Fractions from Germinated Oats (*Avena sativa*)

**DOI:** 10.3390/foods15183241

**Published:** 2026-09-14

**Authors:** Naphatsawan Singhadechachai, Wannaporn Klangpetch, Kridsada Unban, Tabkrich Khumsap, Pornchai Rachtanapun, Apinya Chaikaew, Waritsara Khamjing, Suphat Phongthai, Pipat Tangjaidee

**Affiliations:** 1 Division of Food Science and Technology, Faculty of Agro-Industry, Chiang Mai University, Chiang Mai 50100, Thailand; naphatsawan_sing@cmu.ac.th (N.S.);; 2 Division of Packaging Technology, Faculty of Agro-Industry, Chiang Mai University, Chiang Mai 50100, Thailand

**Keywords:** selenium biotransformation, ultrasonic-assisted extraction, selenium-polysaccharides

## Abstract

Oats are a rich source of bioactive polysaccharides, particularly β-glucan, while selenium is an essential trace element involved in various physiological functions. This study investigated the effects of sodium selenite supplementation during oat germination on selenium accumulation and polysaccharide characteristics, followed by optimization of ultrasound-assisted extraction (UAE). Germination with 150 ppm sodium selenite resulted in a higher extraction yield (10.22%), polysaccharide content (951.04 mg/g extract), and total selenium content in the crude polysaccharide extract (2.91 mg Se/g extract). Response surface methodology identified the optimal UAE conditions as an ultrasonic power of 520 W, an extraction time of 29 min, and a solid-to-liquid ratio of 7.35% (*w*/*v*). FTIR analysis indicated changes in the infrared spectral features of the polysaccharide fraction following selenium enrichment, while the major structural characteristics were generally retained. Selenium enrichment in polysaccharide from germinated oats showed no significant cytotoxicity toward RAW264.7 macrophages. Fractionation using DEAE-52 cellulose chromatography revealed that selenium was unevenly distributed among the polysaccharide fractions, with OP150-3 containing the highest selenium content (0.12 mg Se/g polysaccharide) and exhibiting higher antioxidant activity than the corresponding non-enriched fraction. Overall, selenium-enriched germinated oats show potential as a source of selenium-associated bioactive polysaccharides for functional food and nutraceutical applications.

## 1. Introduction

Selenium (Se) is an essential trace element that cannot be synthesized by the human body and must be obtained from the diet. Selenium intake is closely associated with the maintenance of redox homeostasis, largely through its involvement in antioxidant defense systems [1]. However, the biological effects and safety of selenium depend strongly on its chemical form. Inorganic selenium, such as selenite, generally shows lower bioavailability and a narrower margin between nutritional and toxic doses compared with organic selenium species, which limits its direct application in functional food development.

Biotransformation is an important biological process that converts inorganic selenium (selenate and selenite) into organic forms, such as SeCys, SeMet, and MSeC, through sulfate- and phosphate-related transport and sulfur assimilation pathways. During seed germination, the uptake and biotransformation of Se may promote its association with cellular polysaccharides, potentially altering the structure and physicochemical properties of the polysaccharide matrix. Selenium may be present in forms associated with hydroxyl groups of sugars or incorporated into the polysaccharide structure [2,3]. Therefore, this study hypothesized that Se supplementation during oat germination may influence the association of Se with the polysaccharide matrix, thereby modifying its structural characteristics and potentially enhancing its functional properties.

Oats (*Avena sativa*) are a cereal rich in polysaccharides, particularly β-glucan, which is located mainly in the endosperm cell walls, especially the sub-aleurone layer, with reported levels of approximately 1.8–7.5% [4]. β-Glucan is a bioactive homopolysaccharide composed of glucose units linked by β-(1→3), β-(1→4), and/or β-(1→6) glycosidic bonds. It is commonly found in oats, barley, yeast, and fungi. Although oats may represent a more accessible dietary cereal for nutritional enrichment, they are attractive matrices for investigating the potential effects of selenium enrichment on polysaccharide-associated functional properties [5]. However, the effects of selenium enrichment during oat germination on the characteristics and functional properties of the resulting polysaccharide fractions remain insufficiently understood.

Ultrasound-assisted extraction (UAE) has been widely applied for the extraction of plant polysaccharides because acoustic cavitation generated by ultrasound can promote cell disruption and facilitate the release of polysaccharides [6]. In selenium-enriched plant materials, UAE may facilitate the recovery of selenium-associated polysaccharide fractions. Compared with conventional hot-water extraction, UAE has been reported to improve the extraction yield, extraction rate, and purity of selenium-associated polysaccharide fractions, with increased total selenium contents reported in some extracts. [7].

Therefore, this study aimed to investigate the effects of selenium supplementation during oat germination on selenium accumulation and the functional properties of oat polysaccharides, and to optimize ultrasound-assisted extraction (UAE) conditions for the recovery of selenium-associated polysaccharide fractions. Furthermore, the antioxidant activity and β-glucan content of the purified selenium-associated polysaccharide fraction were evaluated. The findings provide insights into the potential of germination-based selenium biotransformation and UAE for the development of selenium-enriched oat polysaccharide fractions with potential functional applications.

## 2. Materials and Methods

### 2.1. Materials and Chemicals

The oat cultivar SMGOTC21099 was kindly provided by the Samoeng Rice Research Center, Samoeng District, Chiang Mai, Thailand, and stored in a light-resistant container at 4 °C until use.

All chemicals and reagents were of analytical grade and used without further purification. Sodium selenite (Na_2_SeO_3_, 99%), 2,2′-azino-bis(3-ethylbenzothiazoline-6-sulfonic acid) diammonium salt (ABTS, ≥98%), and 2,4,6-tris(2-pyridyl)-s-triazine (TPTZ, ≥99%) were purchased from Sigma-Aldrich (St. Louis, MO, USA). Selenium ICP standard solution and hydrogen peroxide (H_2_O_2_, 30%) were obtained from Merck (Billerica, MA, USA). Ferric chloride hexahydrate (FeCl_3_·6H_2_O, 97%) was purchased from LOBA Chemie (Mumbai, India). Hydrochloric acid (HCl, 37%), sulfuric acid (H_2_SO_4_, 98%), and sodium hydroxide (NaOH) were supplied by RCI Labscan (Bangkok, Thailand). DEAE-cellulose-52 resin (BR grade, CAS No. 9013-34-7) was obtained from Shanghai Macklin Biochemical Co., Ltd. (Shanghai, China). The β-glucan (Mixed Linkage) Assay Kit (K-BGLU) was purchased from Megazyme (Bray, Ireland). All other chemicals and solvents were of analytical grade and obtained from commercial suppliers.

### 2.2. Selenium Biotransformation in Germinated Oats

The oat germination procedure was modified from the method described by Tomasi, Sileoni [8]. Briefly, 50 g of oat seeds were cleaned and soaked in sodium selenite solutions at concentrations of 0, 50, 100, 150, 200, 250, and 300 mg/L using a seed-to-solution ratio of 1:4 (*w*/*v*) for 4 h at 28 °C. After soaking, the selenium solutions were discarded, and the seeds were transferred to germination trays under controlled conditions. During germination, each treatment was irrigated daily with 100 mL of the corresponding sodium selenite solution until approximately 80% of the seedlings had developed roots with a length of about 1 cm. The control group was germinated under identical conditions using distilled water instead of sodium selenite solution.

The germinated oats were harvested when the root length reached approximately 1 cm, according to Gorzolka, Kölling [9]. The samples were thoroughly washed three times with distilled water to remove residual sodium selenite, dried in a vacuum oven at 60 °C for 12 h, and ground into a fine powder. The resulting powders were subsequently used for the determination of crude polysaccharide content and total selenium content. The selenium concentration selected for subsequent extraction optimization and characterization was determined based on the results of selenium accumulation, yield (%) and crude polysaccharide content.

#### Extraction of Crude Polysaccharides

The extraction of crude polysaccharides was modified from the method described by Molaei and Jahanbin [10]. Briefly, 10 g of powdered sample was extracted with distilled water at a solid-to-liquid ratio of 1:15 (*w*/*v*) at 80 °C for 2 h. The extract was centrifuged at 6000 rpm for 15 min, and the supernatant was collected and mixed with four volumes of 95% ethanol (1:4, *v*/*v*). The mixture was stored at 4 °C for 16 h to precipitate crude polysaccharides and subsequently centrifuged at 6000 rpm for 15 min. The resulting precipitate was dried in a vacuum oven at 60 °C for 5 h.

Proteins were removed from the crude polysaccharides using a modified Sevag method as described by Zhou, Wang [11]. Briefly, Sevag reagent (chloroform:n-butanol, 4:1, *v*/*v*) was added to the crude polysaccharide solution and thoroughly mixed in a separatory funnel. The mixture was shaken for 3 min and allowed to stand for 15 min to achieve phase separation. The aqueous phase was collected, re-precipitated with four volumes of 95% ethanol (1:4, *v*/*v*), and kept at 4 °C for 16 h. The precipitate was recovered by centrifugation at 6000 rpm for 15 min, dried in a vacuum oven at 60 °C for 5 h, and stored in airtight containers protected from light at −20 °C until further analysis.

### 2.3. Optimization of Selenium-Associated Crude Polysaccharide Extraction

The extraction conditions for selenium-associated crude polysaccharide were optimized using Response Surface Methodology (RSM) based on a Box–Behnken design (BBD). Three independent variables were investigated: extraction time (10–30 min), ultrasonic power (225–525 W), and solid-to-liquid ratio (5–10%, *w*/*v*). The experimental design and coded levels of the independent variables are presented in Table 1.

Ultrasonic-assisted extraction was performed using an ultrasonic processor (CPX750, 750 W, 20 kHz; Cole-Parmer Instrument Company, Vernon Hills, IL, USA). Throughout the extraction process, the temperature was maintained below 50 °C to minimize thermal degradation of the polysaccharides. Briefly, 10 g of the selected germinated oat powder was mixed with distilled water at the designated solid-to-liquid ratio and subjected to ultrasonic pretreatment under the conditions specified by the experimental design. The pretreated suspension was subsequently extracted in a water bath at 80 °C for 2 h. The extract was centrifuged at 6000 rpm for 15 min, and the supernatant was collected and precipitated with four volumes of 95% ethanol (1:4, *v*/*v*) at 4 °C for 16 h. The resulting precipitate was recovered by centrifugation at 6000 rpm for 15 min, dried in a vacuum oven at 60 °C, sealed in airtight containers, and stored at −20 °C until further analysis.

The experimental data were fitted to the following second-order polynomial equation:

$$Y=\beta_{0}+\sum \beta_{i}X_{i}+\sum \beta_{ii}X_{i}^{2}+\sum \beta_{ij}X_{i}X_{j}$$

where *Y* represents the predicted response; β_0_ is the intercept; β_i_, β_ii_, and β_ij_ are the linear, quadratic, and interaction coefficients, respectively; and X_i_ and X_j_ are the coded independent variables.

Statistical analysis and model generation were performed using Design-Expert software (Version 6.0.10, Stat-Ease Inc., Minneapolis, MN, USA). The complete ANOVA results for the fitted models are provided in the Supplementary Material (Table S1). The optimal extraction conditions were determined by maximizing crude polysaccharide yield, total polysaccharide content, and total selenium content using the desirability function. The predicted optimum conditions were subsequently validated experimentally.

### 2.4. Purification of the Polysaccharide Fraction

Purification of the polysaccharide fraction was performed using DEAE-cellulose-52 anion-exchange chromatography according to the method described by Zhang and Li [12]. Briefly, the crude polysaccharide was dissolved in deionized water and filtered through a 0.45 μm membrane filter to remove insoluble particles. The filtrate was then applied to a DEAE-cellulose-52 column pre-equilibrated with deionized water. The column was sequentially eluted with deionized water followed by 0.1, 0.3, and 0.5 M NaCl solutions at a flow rate of 0.7 mL/min. Eluted fractions were collected at 1 min intervals, and the carbohydrate content of each fraction was monitored by measuring the absorbance at 490 nm. Elution profiles were constructed based on the absorbance values. The collected fractions were subsequently dialyzed against deionized water to remove residual salts and freeze-dried. The purified polysaccharide fractions were stored at −20 °C until further analysis.

### 2.5. Chemical Composition

#### 2.5.1. Extraction Yield

The extraction yield was calculated as the percentage of the dried crude polysaccharide obtained relative to the initial sample weight using the following equation:

Extraction yield (%) = (Weight of dried crude extract (g))/(Initial sample weight (g)) × 100

#### 2.5.2. Total Selenium Content

Total selenium content was determined according to the method described by Mezeyova, Hegedusova [13]. Approximately 200 mg of each sample was transferred into a microwave digestion vessel containing 4 mL of concentrated nitric acid (65% HNO_3_) and 2 mL of hydrogen peroxide (30% H_2_O_2_). The samples were digested using a microwave digestion system (ETHOS UP, Milestone Srl, Sorisole, Italy) at 200 °C for 2 h. After digestion, the digest was diluted to a final volume of 25 mL with deionized water.

Selenium concentration was determined using inductively coupled plasma–mass spectrometry (ICP-MS; Agilent 7900 ICP-MS, Agilent Technologies, Tokyo, Japan) with ^78^Se as the monitored isotope. Quantification was performed using a selenium standard calibration curve, and the results were expressed as micrograms of selenium per gram of dry weight (µg Se/g DW).

Total selenium content was determined in the present study. However, selenium speciation analysis to identify the specific chemical forms of selenium associated with the polysaccharide fraction was not performed, and the presence of covalent selenium–polysaccharide bonds could not be definitively established.

#### 2.5.3. Determination of Polysaccharide with Phenol-Sulfuric Acid Method

Total polysaccharide content was determined using the phenol–sulfuric acid method described by DuBois, Gilles [14] with slight modifications according to Gao, Wang [15]. Briefly, 0.1 g of sample was dissolved in 1 mL of distilled water. An aliquot of 0.5 mL of the sample or glucose standard solution was mixed with 2.5 mL of concentrated sulfuric acid (96%) and 0.5 mL of 5% phenol. Subsequently, 200 µL of the reaction mixture was transferred to a 96-well microplate, and the absorbance was measured at 490 nm using a microplate spectrophotometer. Total polysaccharide content was calculated from a glucose standard calibration curve and expressed as milligrams of glucose equivalent per gram of sample (mg GE/g extract).

### 2.6. Antioxidant Activity

#### 2.6.1. ABTS Assay

The ABTS radical scavenging activity was determined according to the method described by Arnao and Cano [16]. The stock ABTS•^+^ solution was prepared by mixing 7.4 mM ABTS with 2.45 mM potassium persulfate (K_2_S_2_O_8_) 2.45 mM at a ratio of 1:1 (*v*/*v*). The mixture was kept in the dark at 4 °C for 16 h to allow complete radical formation. The resulting ABTS•^+^ solution was then diluted with distilled water to obtain an absorbance of 0.7 ± 0.02 at 734 nm. The decrease in absorbance was measured after sample addition, and the antioxidant activity was expressed as Trolox equivalent antioxidant capacity (TEAC, µmol TE/g sample) using a Trolox standard calibration curve.

#### 2.6.2. FRAP Assay

Ferric reducing antioxidant power (FRAP) was determined according to the method of Benzie and Strain [17]. The FRAP reagent was freshly prepared by mixing 10 mM 2,4,6-Tripyridyl-s-Triazine (TPTZ) in 40 mM hydrochloric acid (HCl), 20 mM FeCL_3_.6H_2_O in distilled water and acetate buffer pH 3.6 at a ratio of 1:1:10 (*v*/*v*/*v*). Subsequently, 20 µL of sample was mixed with 180 µL of FRAP reagent in a 96-well microplate and incubated for 5 min at room temperature. The absorbance was measured at 595 nm. A standard calibration curve was constructed using Trolox, and the antioxidant capacity was expressed as Trolox equivalent antioxidant capacity (TEAC, µmol TE/g sample).

#### 2.6.3. ORAC Assay

The oxygen radical absorbance capacity (ORAC) assay was performed according to the method of Giordano, Morales-Tapia [18] with slight modifications. Samples and Trolox standard (6.25–50 µM) were diluted in buffer solution (phosphate buffer, pH 7.4). An aliquot of 35 µL of each standard was mixed with 50 µL of Fluorescein solution, followed by the addition of 50 µL of AAPH to initiate peroxyl radical generation. The reaction mixture was incubated at 37 °C, and fluorescence was recorded every 5 min for 180 min using a microplate reader (excitation 485 nm; emission 520 nm). The antioxidant activity was calculated from the area under the curve (AUC) of fluorescence decay relative to the Trolox calibration curve. Results were expressed as micromoles of Trolox equivalents per gram of sample (µmol TE/g).

### 2.7. Determination of β-Glucan Content

The β-glucan content of the purified polysaccharide fractions was determined using a Mixed Linkage β-Glucan Assay Kit (K-BGLU; Megazyme, Bray, Ireland) according to the manufacturer’s instructions. Briefly, samples were hydrolyzed with lichenase, followed by enzymatic hydrolysis with β-glucosidase to convert β-glucan into glucose. The released glucose was quantified colorimetrically using a glucose oxidase/peroxidase (GOPOD) reagent, and the absorbance was measured at 510 nm using a microplate reader. The β-glucan content was calculated according to the manufacturer’s protocol and expressed as a percentage of dry weight (%).

### 2.8. Cell Viability Assay

The cytotoxicity of the purified polysaccharide fractions was evaluated using RAW264.7 murine macrophages (ATCC CCL-185). Cells were cultured in Dulbecco’s Modified Eagle Medium (DMEM) supplemented with 10% fetal bovine serum (FBS) and 1% penicillin–streptomycin at 37 °C in a humidified incubator containing 5% CO_2_.

Cells were seeded into 96-well plates and incubated for 24 h before treatment with purified polysaccharide samples at the indicated concentrations. After 24 h of incubation, cell viability was determined using the MTT assay according to the method described by Hankittichai, Buacheen [19]. Briefly, MTT solution was added to each well and incubated for 4 h. The resulting formazan crystals were dissolved in dimethyl sulfoxide (DMSO), and the absorbance was measured at 570 nm using a microplate reader. Cell viability was expressed as a percentage relative to the untreated control.

### 2.9. Fourier-Transform Infrared (FTIR) Spectroscopy

Approximately 3 mg of each purified polysaccharide sample was thoroughly mixed with potassium bromide (KBr) powder and compressed into pellets approximately 1 mm in thickness. Fourier-transform infrared (FTIR) spectra were recorded using an FT/IR-4700 spectrophotometer (JASCO Corporation, Tokyo, Japan) equipped with a TGS detector over the wavenumber range of 4000–400 cm^−1^ at a spectral resolution of 4 cm^−1^ with 32 accumulated scans. The spectra were analyzed to identify the characteristic functional groups of the purified polysaccharides.

### 2.10. Statistical Analysis

All experiments were performed in triplicate, and the results are presented as the mean ± standard deviation (SD). Statistical analyses were performed using SPSS Statistics version 17.0 (SPSS Inc., Chicago, IL, USA). Differences among fractions within the same treatment were evaluated by one-way analysis of variance (ANOVA) followed by Duncan’s multiple range test. Response surface methodology (RSM) and regression analyses were carried out using Design-Expert software (Version 6.0.10, Stat-Ease Inc., Minneapolis, MN, USA). Differences were considered statistically significant at *p* < 0.05.

## 3. Results and Discussion

### 3.1. Investigation of the Selenium Biotransformation Process in Oats

Selenium supplementation significantly affected oat germination in a concentration-dependent manner. Root elongation was progressively inhibited at selenium concentrations exceeding 200 ppm (Figure S1), indicating that excessive selenium supplementation negatively affected seedling growth. Similar inhibitory effects have been reported in wheat seedlings exposed to excessive selenium, where high selenium concentrations disrupted normal cellular metabolism and suppressed plant development [20]. The growth inhibition may be associated with selenium toxicity, which can interfere with physiological processes and metabolic homeostasis when selenium accumulates beyond the level required for normal plant growth [21]. Selenite is primarily absorbed through phosphate transporters and aquaporins, facilitating its translocation into seed tissues during germination [22,23].

**Figure 1:**
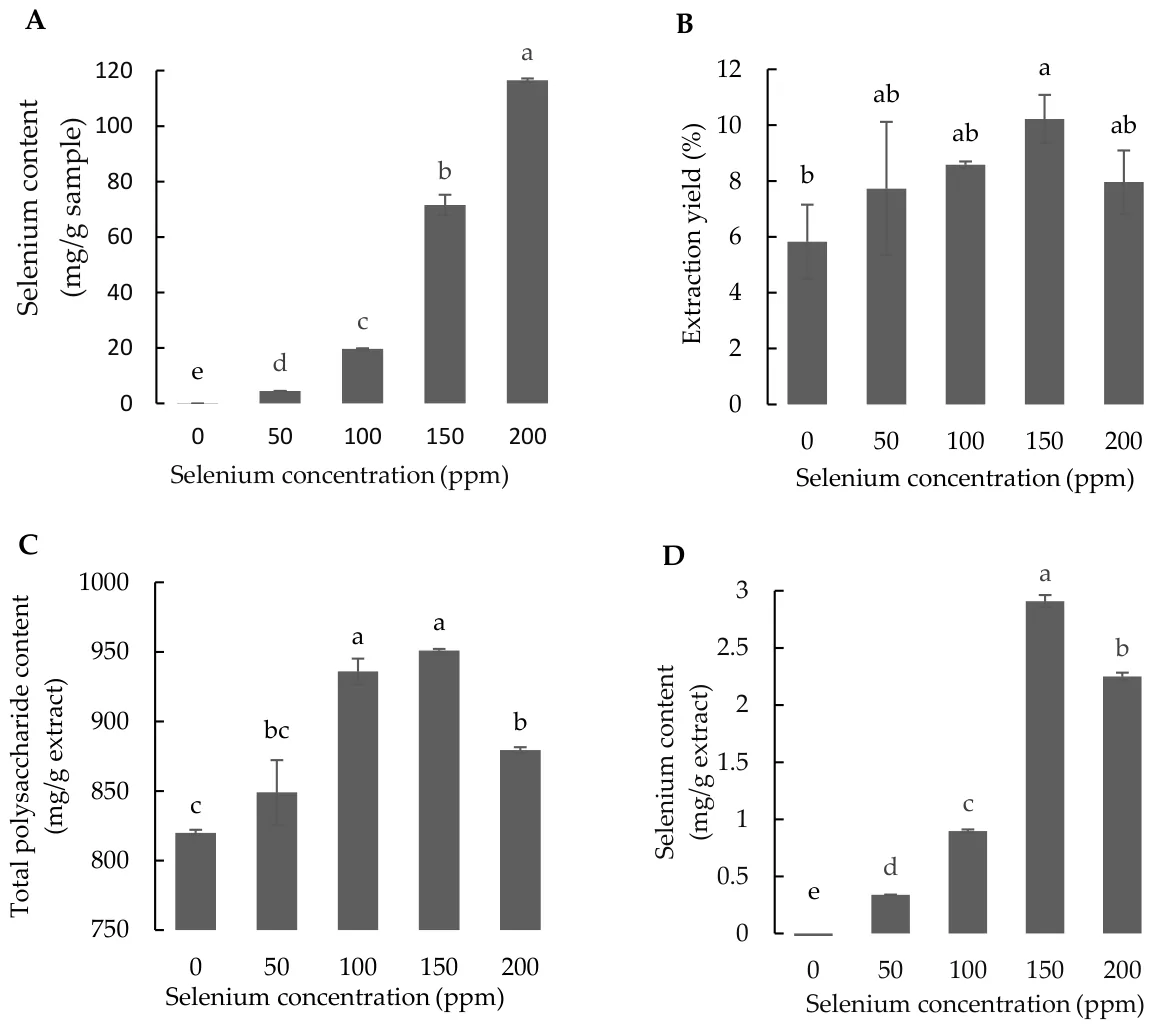
Selenium and polysaccharide properties of germinated oats treated with different sodium selenite concentrations. (**A**) Selenium content in oat powder by ICP-MS. (**B**) HWE extraction yield (%). (**C**) Total polysaccharide content in HWE extract (mg/g extract). (**D**) Selenium content in HWE extract by ICP-MS (mg Se/g extract). Note: Values are expressed as mean ± standard deviation (*n* = 3). Different lowercase letters above the bars indicate significant differences among treatments according to Duncan’s multiple range test (*p* < 0.05). Means sharing the same letter are not significantly different.

As shown in Figure 1A, selenium accumulation in germinated oat powder increased significantly with increasing sodium selenite supplementation, reaching a maximum of 116.91 mg Se/g sample at 200 ppm. This concentration-dependent increase demonstrates the capacity of germinating oats to accumulate selenium. During germination, absorbed selenite can be metabolically transformed through sulfur metabolism into organic selenium compounds, including selenocysteine and selenomethionine [24,25]. These findings demonstrate that germinated oats exhibit a high selenium accumulation capacity and therefore represent a promising source of organically bound selenium for functional food applications [26].

Following selenium accumulation, the effects of selenium supplementation on the extraction yield and total polysaccharide content obtained by hot-water extraction (HWE) were evaluated (Figure 1B,C). Selenium supplementation at 150 ppm resulted in the highest extraction yield (10.22%), whereas higher selenium concentrations resulted in a gradual decrease. Total polysaccharide content showed a similar trend, with the highest values at 100 ppm (935.88 mg/g extract) and 150 ppm (951.04 mg/g extract). The reduction in extraction yield and total polysaccharide content at higher selenium concentrations may be associated with selenium toxicity and its adverse effects on normal physiological and metabolic processes during germination [27,28]. The concurrent inhibition of root elongation at higher selenium concentrations (Figure S1) further supports the negative effects of excessive selenium supplementation on oat development. However, the specific biochemical mechanism underlying this response cannot be established from the present data.

The selenium content of the crude extracts obtained by hot water extraction also varied significantly among treatments (Figure 1D). The highest selenium content (2.91 mg Se/g extract) was detected in oats supplemented with 150 ppm sodium selenite, which was significantly higher than that obtained at 200 ppm (2.25 mg Se/g extract) (*p* < 0.05). Notably, total selenium accumulation in oat tissue was highest at 200 ppm, whereas selenium content in the crude polysaccharide extract was highest at 150 ppm. This difference suggests that total selenium accumulation in oat tissue did not directly correspond to selenium association with the extractable polysaccharide fraction. The lower selenium content in the crude extract at 200 ppm may reflect differences in selenium distribution or metabolism and the lower amount of polysaccharide recovered at this concentration. The specific molecular form or binding mode of selenium cannot be established from the present measurements alone.

Overall, selenium supplementation resulted in a concentration-dependent response in germinating oats. Although total selenium accumulation was highest at 200 ppm, excessive supplementation was accompanied by reduced root growth and lower crude polysaccharide extraction yield and total polysaccharide content. Among the tested concentrations, 150 ppm provided the most favorable balance, yielding the highest crude polysaccharide extraction yield (10.22%), high total polysaccharide content (951.04 mg/g extract), and the highest selenium content in the crude polysaccharide extract (2.91 mg Se/g extract). Therefore, oats germinated with 150 ppm sodium selenite were selected for subsequent extraction, purification, and structural characterization of the selenium-associated crude polysaccharide.

### 3.2. Optimization of Selenium-Containing Polysaccharide Extraction Using Ultrasound-Assisted Extraction (UAE)

**Figure 2:**
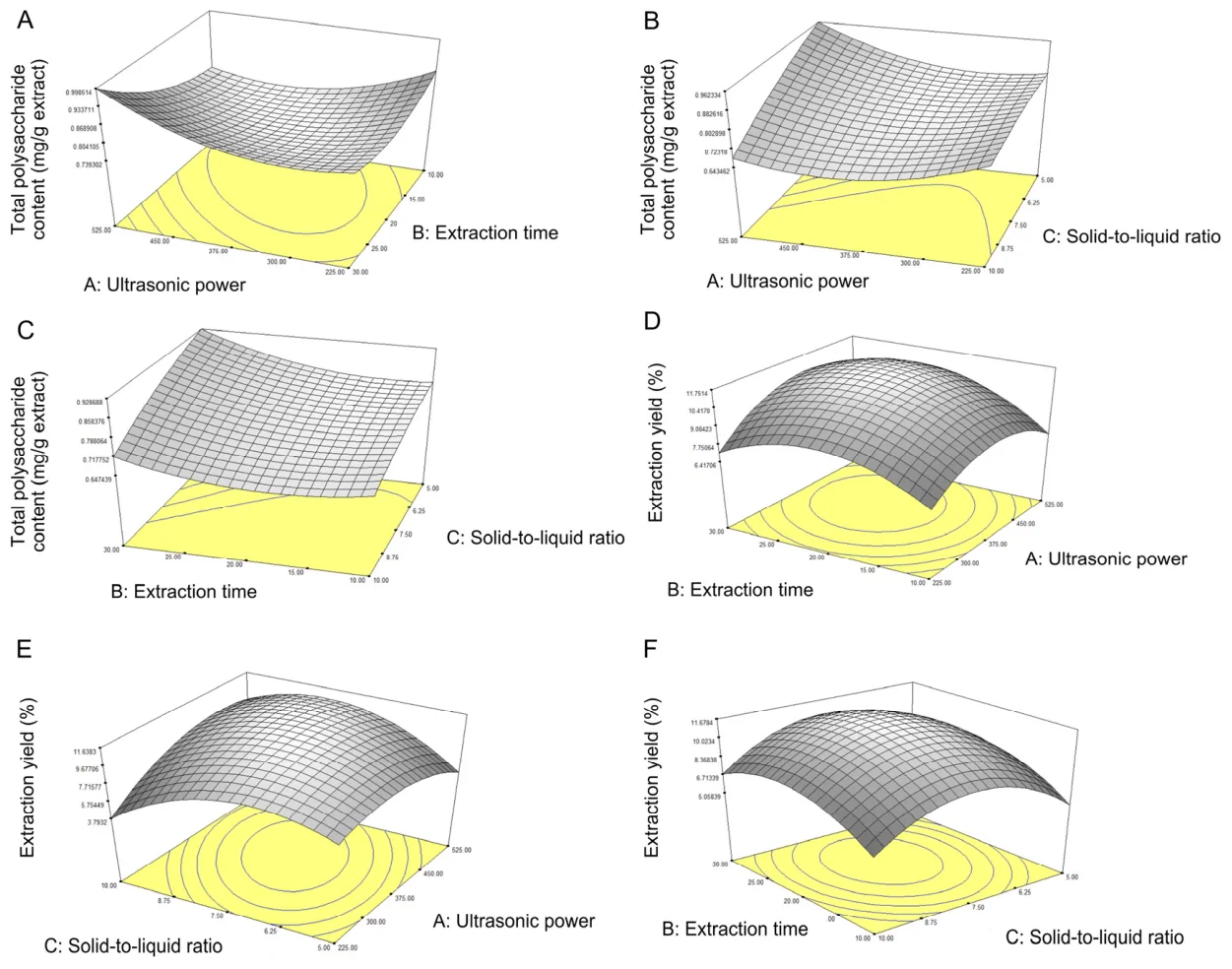
Response surface plots illustrating the effects of ultrasonic power, extraction time, and solid-to-liquid ratio on polysaccharide content (**A**–**C**) and extraction yield (**D**–**F**). (**A**,**D**) Ultrasonic power and extraction time; (**B**,**E**) ultrasonic power and solid-to-liquid ratio; and (**C**,**F**) extraction time and solid-to-liquid ratio.

A 15-run Box–Behnken design (BBD) was employed to optimize ultrasound-assisted extraction (UAE) of polysaccharides from selenium-enriched germinated oats. The experimental design matrix and corresponding response values, including extraction yield (%), total polysaccharide content (mg/g extract), and selenium content (mg Se/g extract), are summarized in Table 1. The response surface plots (Figure 2A–C) showed that ultrasonic power, extraction time, and the solid-to-liquid ratio collectively affected polysaccharide extraction. As shown in Figure 2A, increasing ultrasonic power and extraction time within the investigated range enhanced total polysaccharide content. This improvement is attributed to intensified acoustic cavitation, which disrupts plant cell walls, weakens the interactions between polysaccharides and structural proteins, and facilitates the release of intracellular polysaccharides into the extraction medium. In addition, cavitation-generated shear forces may induce moderate depolymerization, reducing molecular weight and improving polysaccharide solubility, thereby increasing the measurable polysaccharide content in the extract [29,30].

In contrast, Figure 2B,C demonstrated that increasing the solid-to-liquid ratio, corresponding to a lower solvent volume at a constant sample mass, significantly reduced total polysaccharide content regardless of extraction time. This effect is primarily associated with reduced mass transfer efficiency. Although a lower solvent volume theoretically increases ultrasonic energy density, insufficient liquid restricts acoustic wave propagation and suppresses cavitation-induced micro-streaming, thereby limiting solvent penetration into the plant matrix. Furthermore, according to Fick’s second law, the reduced solvent volume accelerates solvent saturation and decreases the concentration gradient driving polysaccharide diffusion, ultimately limiting extraction efficiency [31]. Excessive sonication may also promote polysaccharide degradation, which adversely affects structural integrity and reduces the functional properties of the extracted polysaccharides [32].

Among the investigated factors, the solid-to-liquid ratio exerted the greatest influence on extraction yield, followed by extraction time and ultrasonic power (Figure 2D–F). As illustrated in the response surface plots, extraction yield increased with increasing extraction time and solvent volume, reflecting enhanced solvent penetration and improved mass transfer from the plant matrix [33]. However, when extraction time exceeded approximately 20 min and the solid-to-liquid ratio exceeded 7.5% (*w*/*v*), extraction yield reached a plateau or declined slightly. This phenomenon is likely attributable to reduced effective ultrasonic energy density at excessive solvent volumes, which weakens cavitation intensity, together with heat accumulation during prolonged sonication that may promote degradation of extracted compounds [34]. Similar trends have been reported for ultrasound-assisted extraction of polysaccharides from *Flammulina velutipes* stipe, where extraction efficiency increased under moderate extraction conditions but declined beyond the optimum range [35]. These findings highlight that the interaction among ultrasonic power, extraction time, and the solid-to-liquid ratio is critical for maximizing polysaccharide recovery.

The relationships between the extraction variables and total polysaccharide content were evaluated using the fitted quadratic model. Among the investigated variables, the solid-to-liquid ratio (C) and the quadratic effect of ultrasonic power (A^2^) significantly influenced total polysaccharide content (*p* < 0.05). The quadratic models for total polysaccharide content and extraction yield were statistically significant (*p* < 0.05), indicating a good fit between the predicted and experimental values. In contrast, the model for total selenium content was not statistically significant (*p* = 0.36), indicating that ultrasonic power, extraction time, and solid-to-liquid ratio did not significantly affect the measured selenium content within the investigated experimental range. This result may be attributed to the same initial selenium load from germinated oat under the same selenium supplementation condition (150 ppm) as the original material for all extraction experiments. Therefore, the selenium content of the starting material was relatively consistent across the experimental runs, and variation in the UAE parameters within the investigated range did not produce a measurable change in the amount of selenium recovered in the extract. The total selenium content of the extract may depend more strongly on the selenium level of the starting material than on the UAE conditions investigated in this study. In contrast, the significant effects observed for polysaccharide content and extraction yield indicate that the UAE conditions influenced the recovery of polysaccharide material rather than the selenium concentration of the extracts. The complete ANOVA results for the fitted models are provided in the Supplementary Material (Table S1) to ensure statistical and methodological transparency. Consequently, only total polysaccharide content and extraction yield were selected as optimization responses for determining the optimal ultrasonic-assisted extraction conditions. The fitted quadratic polynomial equations describing total polysaccharide content and crude polysaccharide yield are shown in Equations (1) and (2), respectively.

*Total polysaccharide content = 1.13634 − 0.002188A − 0.024599B + 0.088063C + 0.00000344699A^2^ + 0.000497284B^2^ − 0.00295478C^2^ + 0.0000427083(AB) − 0.000153898(AC) − 0.000932133(BC)* (1)

*E xtraction yield (%) = −28.04905 + 0.028423A + 0.86328B + 6.39941C − 0.0000885279A^2^ − 0.021624B^2^ − 0.53723C^2^ + 0.000355259(AB) + 0.00469971(AC) − 0.00565803(BC)* (2)

Response surface methodology (RSM) analysis further demonstrated that the quadratic model for extraction yield showed excellent predictive performance (R^2^ = 0.9581), indicating a strong agreement between the model and the experimental data (Table 2). Regression analysis identified extraction time and the solid-to-liquid ratio as the primary linear factors influencing extraction yield, while significant interaction terms (*p* < 0.05) confirmed that the extraction variables acted synergistically to determine extraction efficiency.

Based on the fitted RSM models, the optimal extraction conditions were predicted to be an ultrasonic power of 520 W, an extraction time of 29 min, and a solid-to-liquid ratio of 7.35% (*w*/*v*). Validation experiments performed under these conditions demonstrated good agreement between the predicted and experimental values, with relative errors of 4.35%for total polysaccharide content and 1.41% for crude polysaccharide extraction yield (Table 2). The low prediction errors confirm the robustness and predictive capability of the developed models, indicating that the optimized ultrasonic-assisted extraction conditions are suitable for efficient recovery of selenium-associated crude polysaccharide.

### 3.3. Toxicity of Selenium-Containing Polysaccharide on RAW264.7 Macrophage Cells

**Figure 3:**
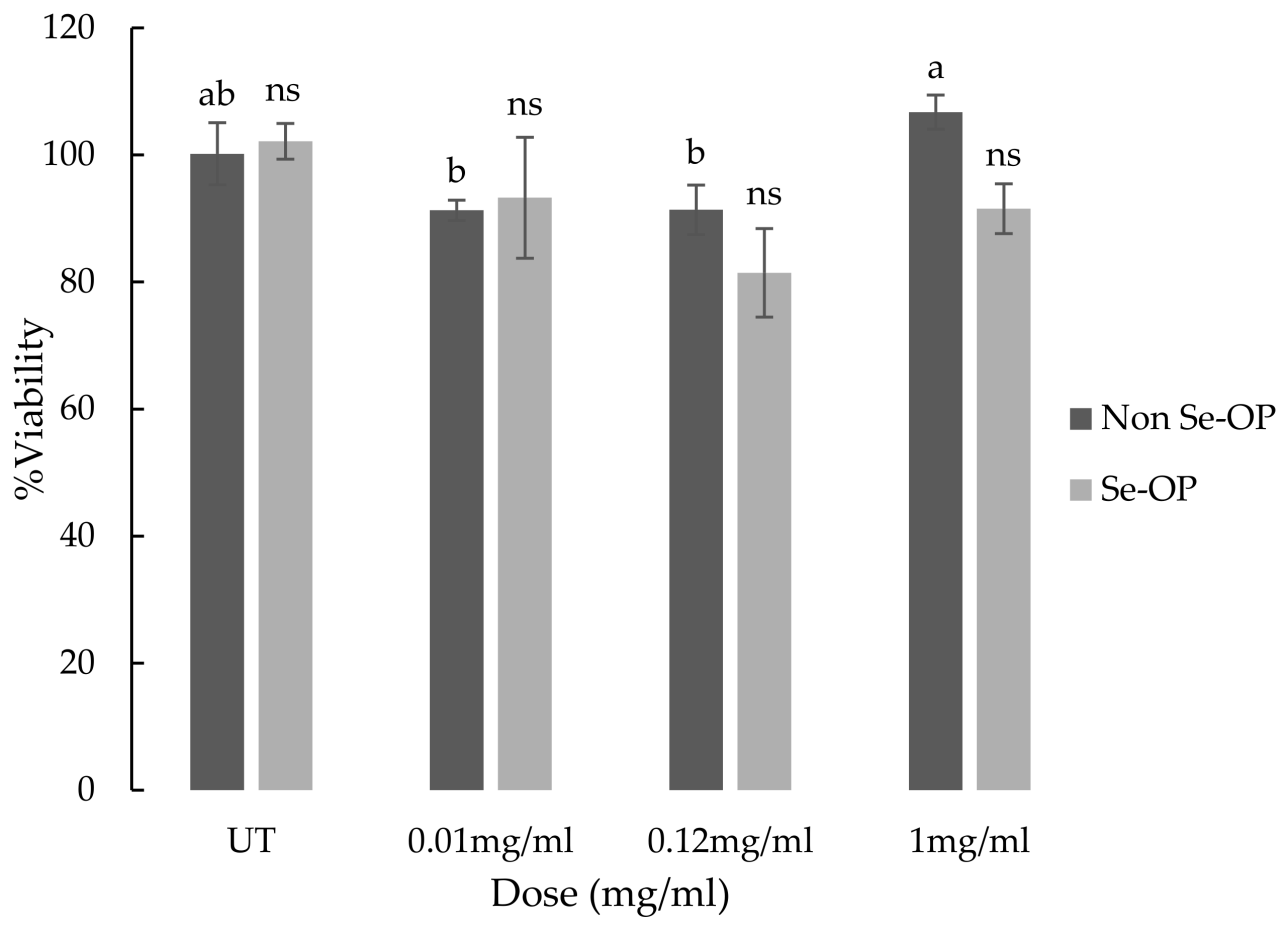
Effect of crude polysaccharides at different concentrations on cell viability. Note: Data are presented as mean ± SD (*n* = 3); statistical analysis revealed no significant differences between the treated samples and the untreated (UT) controls (*p* > 0.05). Different lowercase letters indicate statistically significant differences among groups (*p* < 0.05) and ns indicate non-significant difference among groups (*p* > 0.05).

The cytotoxicity of crude polysaccharides was evaluated in RAW264.7 macrophages to assess their safety and suitability for subsequent bioactivity studies. Cell viability was determined after 24 h of treatment using the MTT assay. As shown in Figure 3, treatment with both selenium-associated crude polysaccharide and non- selenium-associated crude polysaccharide at concentrations of 0.01, 0.05, and 0.1 mg/mL did not significantly affect the viability of RAW264.7 macrophages compared with the untreated control (*p* > 0.05). These findings indicate that neither polysaccharide sample exhibited apparent cytotoxicity within the tested concentration range, demonstrating their good biocompatibility toward RAW264.7 cells.

The favorable cytocompatibility of the selenium-associated crude polysaccharide may be associated with selenium biotransformation during plant germination. In this process, inorganic selenium is converted into organic selenium-containing compounds with higher bioavailability and lower toxicity than inorganic selenium species [2]. Selenium biofortification has also been reported to promote the accumulation of nutritionally beneficial organic selenium compounds that can be more readily utilized by biological systems [36]. Although selenium speciation was not determined in the present study, the absence of cytotoxicity observed in RAW264.7 macrophages is consistent with these previous findings. In comparative cell viability assays, inorganic selenium (such as sodium selenite) typically exhibits high cytotoxicity toward RAW264.7 cells at concentrations as low as 2 mg/mL, often causing significant antiproliferative effects exceeding 20% growth inhibition. In contrast, organically bound selenium in polysaccharides generally retains high cell viability (>90%) across broad concentration ranges (50–800 mg/mL) and can even promote macrophage proliferation and functional activation without inducing cell death [37,38]. Collectively, these findings further support the safety of selenium-containing polysaccharides at the concentrations tested and provide a basis for subsequent investigations of their biological activities.

### 3.4. Purification and Chemical Composition of Polysaccharide Fractions from Selenium-Enriched Oats

**Figure 4:**
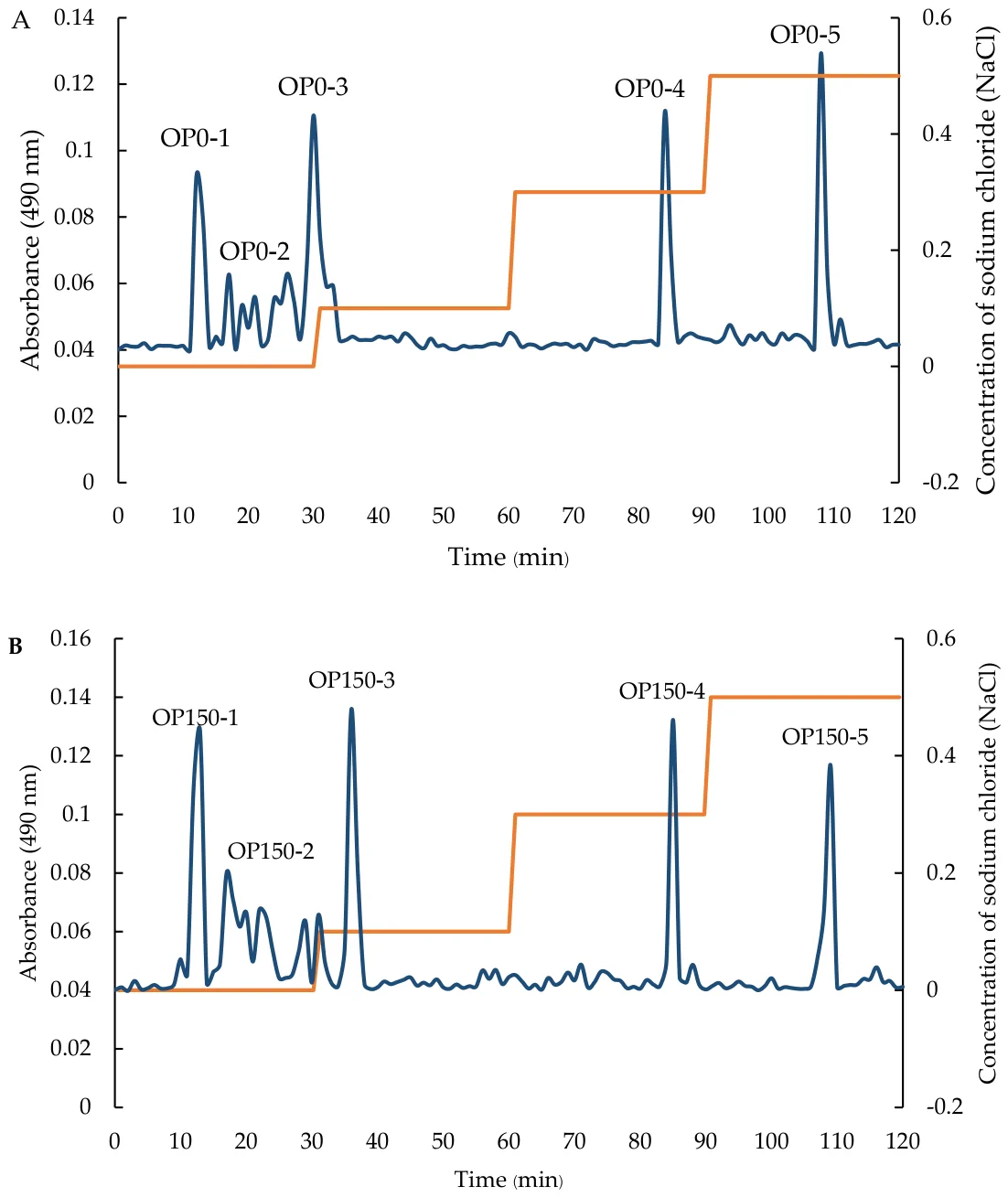
Elution profiles of crude polysaccharides fractionated by DEAE-52 cellulose anion-exchange chromatography. (**A**) Non-selenium-enriched oats; (**B**) selenium-enriched oats germinated with 150 ppm sodium selenite. The blue line represents absorbance, while the orange line represents the NaCl concentration.

Crude polysaccharides extracted from both control and selenium-enriched oats were fractionated by DEAE-cellulose-52 anion-exchange chromatography. The two samples exhibited comparable elution profiles, each consisting of five major polysaccharide fractions, designated OP0-1 to OP0-5 for the control (Figure 4A) and OP150-1 to OP150-5 for the polysaccharide fraction obtained from selenium-enriched oats (Figure 4B).

Selenium analysis revealed an uneven distribution among the fractions, with OP150-3 containing the highest selenium content at 0.12 mg Se/g polysaccharide (Table 3). Selenium was also detected in OP150-4 at 0.02 mg Se/g polysaccharide, whereas selenium was not detected in OP150-1, OP150-2, or OP150-5. No selenium was detected in any of the corresponding fractions from the control sample.

The β-glucan content was subsequently determined in both the crude polysaccharides and the individual fractions obtained after DEAE-52 chromatography (Table 3). Overall, the crude polysaccharide and individual fractions obtained from selenium-treated oats generally exhibited higher β-glucan contents than the corresponding control samples. The crude polysaccharide extracted from selenium-enriched oats contained 4.79 g/100 g β-glucan, compared with 3.09 g/100 g in the control sample. Likewise, fractions OP150-1 to OP150-5 contained 4.56, 4.61, 4.00, 3.83, and 2.97 g/100 g dry weight, respectively, whereas the corresponding control fractions contained 2.79, 2.88, 3.86, 3.02, and 2.85 g/100 g dry weight, respectively. The higher β-glucan contents generally observed in the selenium-treated samples may indicate that selenium supplementation influenced carbohydrate metabolism during germination. Selenium has been reported to enhance α-amylase activity, thereby accelerating starch degradation and increasing the availability of soluble carbohydrates, which may subsequently affect β-glucan biosynthesis or retention in germinated grains [39]. Although the underlying mechanism was not investigated in the present study, these findings are consistent with previous reports indicating that selenium supplementation can modify carbohydrate metabolism and consequently alter the polysaccharide composition of germinated cereals.

Because β-glucan is a major soluble polysaccharide component of oats, the observed differences in β-glucan content may contribute to differences in the composition and physicochemical characteristics of the oat polysaccharide matrix. These differences in polysaccharide composition may partly explain the variation in the characteristics of the fractions obtained after DEAE-52 chromatography. However, further structural analysis would be required to determine whether selenium enrichment directly alters the molecular structure or organization of the β-glucan-rich polysaccharide matrix.

The elution behavior of the DEAE-52 fractions may provide further insight into the distribution of selenium among the polysaccharide fractions. The crude polysaccharide was loaded onto the DEAE-52 column in 14 consecutive runs at 0.15 g per run, giving a total loading of 2.10 g. The total mass recovered from OP150-1 to OP150-5 was 0.69 g, corresponding to an overall fraction recovery of 32.67% based on the initial crude polysaccharide mass loaded onto the column.

DEAE-cellulose is an anion-exchange matrix that interacts with negatively charged polysaccharides, and polysaccharides with stronger ionic interactions generally require higher ionic strength for elution [40]. The elution of OP150-3, OP150-4, and OP150-5 at relatively higher NaCl concentrations suggests that these fractions may contain polysaccharide components with relatively stronger ionic interactions with the DEAE matrix or different charge characteristics compared with fractions eluted at lower salt concentrations. The differential selenium distribution across fractions may be consistent with the mechanism of selenium binding to oxygen nucleophiles on the polysaccharide backbone. Because DEAE anion-exchange retention is governed principally by anionic group density, and elution ionic strength increases with the acidic character of a fraction, the observed correlation between selenium content and NaCl concentration required for elution provides indirect but consistent evidence. These findings indicate a structure-selective mode of selenium binding, involving uronic-acid carboxyl groups, sulfate esters, and hydroxyl moieties on neutral glucan backbones. Notably, the covalent nature of the resulting Se-ester linkage introduces additional anionic character to the polysaccharide, a property that is consistent with, rather than confounding to, the charge-based separation achieved during ion-exchange chromatography. The relatively high selenium content observed in OP150-3 and OP150-4 may therefore reflect an association of selenium with the polysaccharide components present in these fractions. However, because selenium speciation and the specific selenium-binding mode were not determined in the present study, this interpretation remains tentative and cannot establish whether selenium itself contributed directly to the interaction with the DEAE matrix.

During aqueous extraction and ethanol precipitation, polysaccharides are recovered as the precipitated fraction, whereas water-soluble selenium species not associated with the polysaccharide fraction may remain in the supernatant and be removed during subsequent purification. This may partly explain the lower selenium content in the purified polysaccharide fractions compared with the germinated oat powder. However, the selenium contents of the washing waters and discarded fractions were not quantified; therefore, the extent of selenium removal cannot be determined.

### 3.5. Antioxidant Activity and Cytotoxicity on RAW264.7 Macrophage Cells of the Selenium-Associated Polysaccharide Fraction

**Figure 5:**
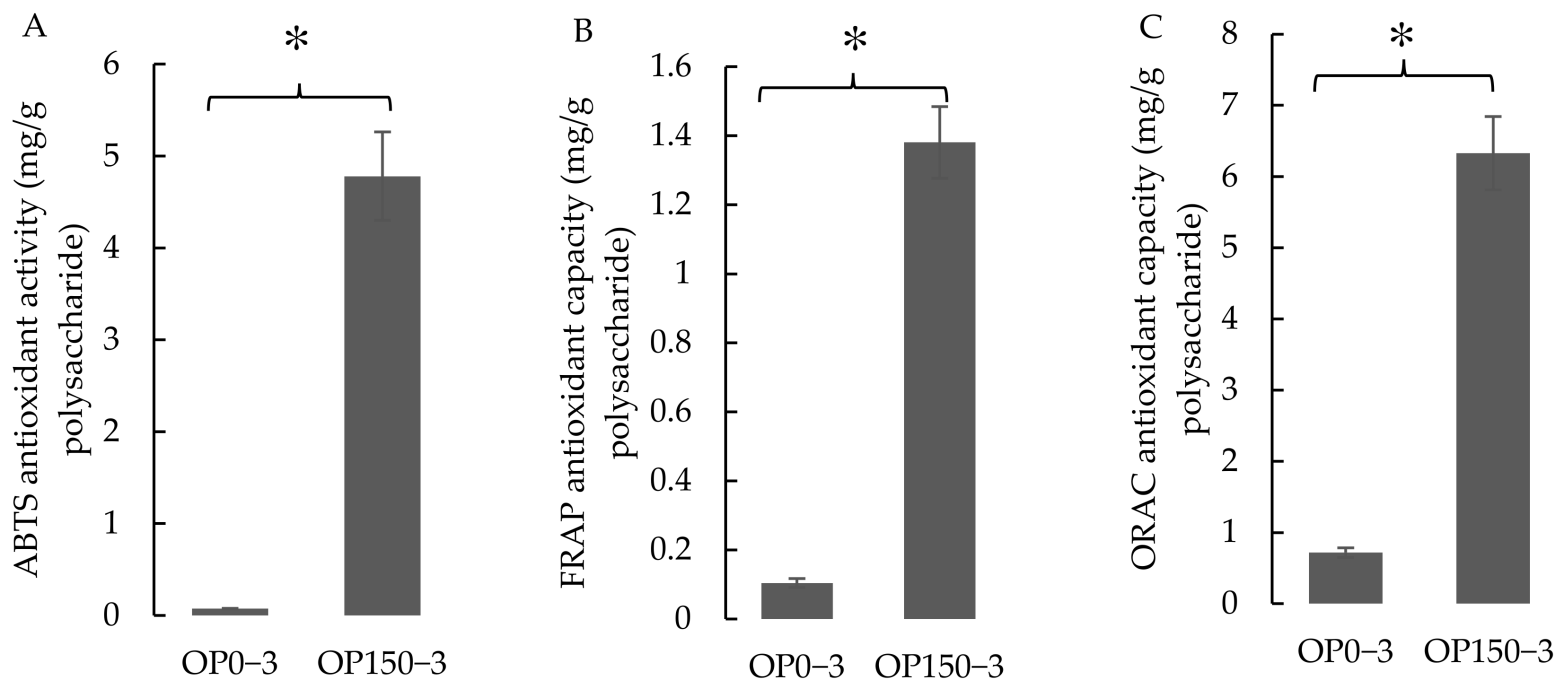
Antioxidant activities of DEAE-52 OP-3 fraction from oat polysaccharide (OP) with selenium fortification (0 and 150 ppm Se) determined by (**A**) ABTS, (**B**) FRAP, and (**C**) ORAC assays. Bars represent mean ± SD (*n* = 3). *p*-values indicate differences between groups (Welch’s two-tailed *t*-test). An asterisk (*) indicates a statistically significant difference between groups (*p* < 0.05).

Antioxidant activities of the selenium-associated polysaccharide fraction and the corresponding non-selenium-associated polysaccharide fraction were evaluated, as shown in Figure 5. Compared with the control fraction OP0-3, the selenium-enriched fraction OP150-3 demonstrated markedly higher antioxidant capacities. The ABTS, FRAP, and ORAC values increased from 0.07, 0.10, and 0.71 mg TE/g polysaccharide in OP0-3 to 4.78, 1.38, and 6.32 mg TE/g polysaccharide in OP150-3, respectively. These results demonstrate that the selenium-enriched OP150-3 fraction exhibited substantially greater antioxidant capacity than the corresponding control fraction, highlighting its potential functional advantage.

The enhanced antioxidant activity may be related to differences in the chemical composition and structural characteristics of the polysaccharide fractions following selenium enrichment. ABTS and FRAP are commonly considered electron-transfer-based assays that reflect reducing capacity, whereas ORAC is primarily associated with hydrogen atom transfer-mediated radical scavenging [41]. Accordingly, the antioxidant activity of selenium-enriched polysaccharides may be attributed, at least in part, to their ability to donate electrons and/or hydrogen atoms, thereby quenching free radicals and inhibiting radical-mediated chain reactions [42]. Selenium-associated polysaccharides have also been reported to exhibit antioxidant activity, which may be influenced by the presence and chemical environment of selenium-containing groups [43]. The substantially higher ABTS, FRAP, and ORAC activities observed for OP150-3 indicate a clear functional difference between the selenium-enriched and control polysaccharide fractions. However, because selenium speciation and the specific selenium-binding model were not determined in the present study, the contribution of a particular selenium species or selenium–polysaccharide interaction to the enhanced antioxidant activity of OP150-3 cannot be definitively established.

**Figure 6:**
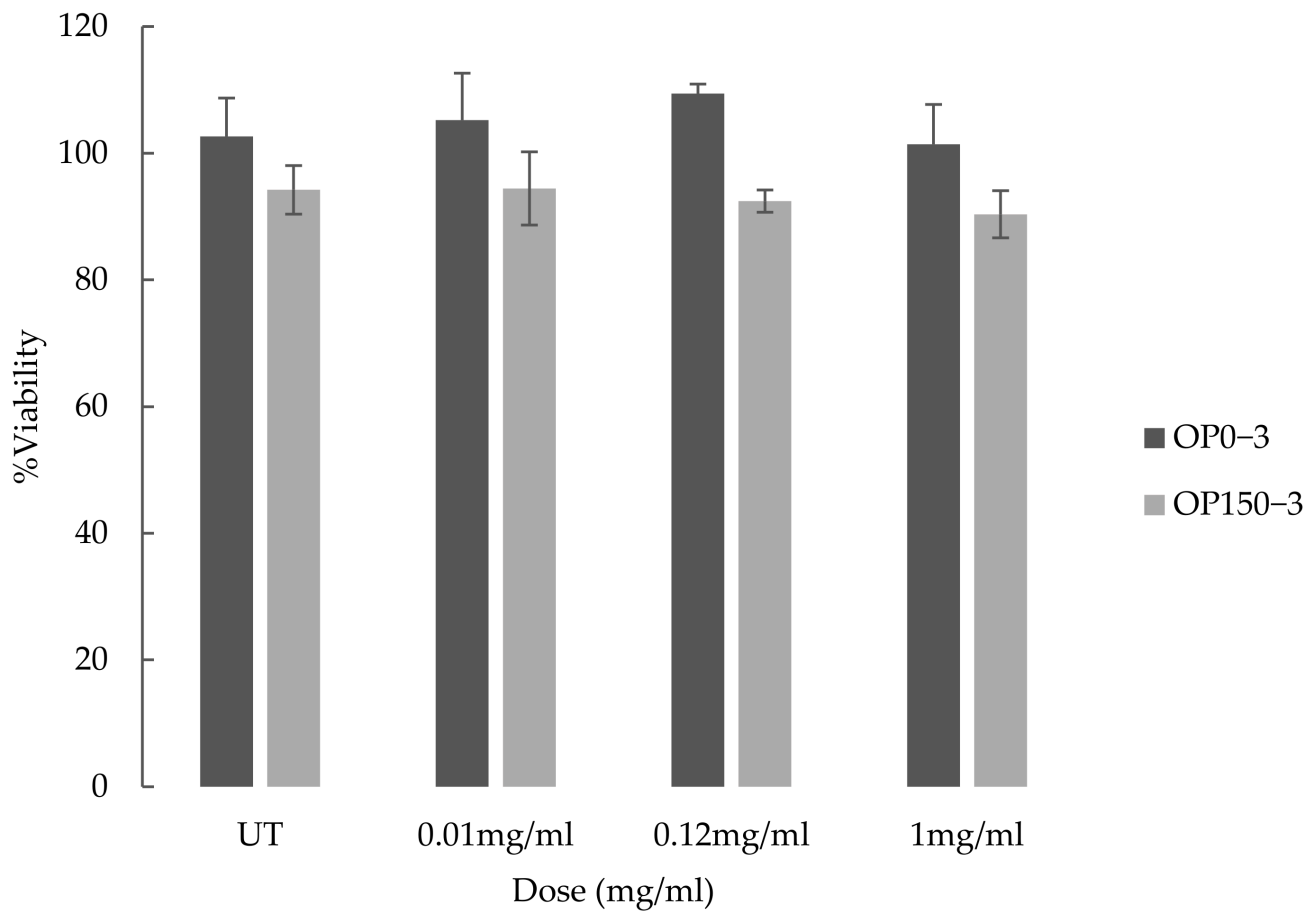
Cytotoxic effects of OP0-3 and Se-enriched OP150-3 on cell viability at different concentrations. UT, untreated control. Note: Data are presented as mean ± SD (*n* = 3); statistical analysis revealed no significant differences between the treated samples and the untreated (UT) controls (*p* > 0.05).

The cytotoxicity of the purified polysaccharide fractions was evaluated using OP0-3 and selenium-enriched OP150-3, which corresponded to the third fractions obtained from the non-selenium-enriched and Se-enriched samples, respectively. As shown in Figure 6, treatment with OP0-3 at concentrations ranging from 0.01 to 1 mg/mL did not significantly affect cell viability compared with the control group. Similarly, selenium-enriched OP150-3 did not significantly affect cell viability at any of the tested concentrations. Although a decreasing trend in cell viability was observed at the highest concentration of Se-enriched OP150-3 (1 mg/mL), the difference was not statistically significant. This finding is consistent with the non-toxic profile of the selenium-containing polysaccharide reported in Section 3.3, further supporting the safety of selenium-containing polysaccharides derived from oat sprouts. These results provide a basis for further toxicological evaluation and support their potential bioactive applications.

### 3.6. FTIR Characterization of Selenium-Associated Polysaccharide Fraction

**Figure 7:**
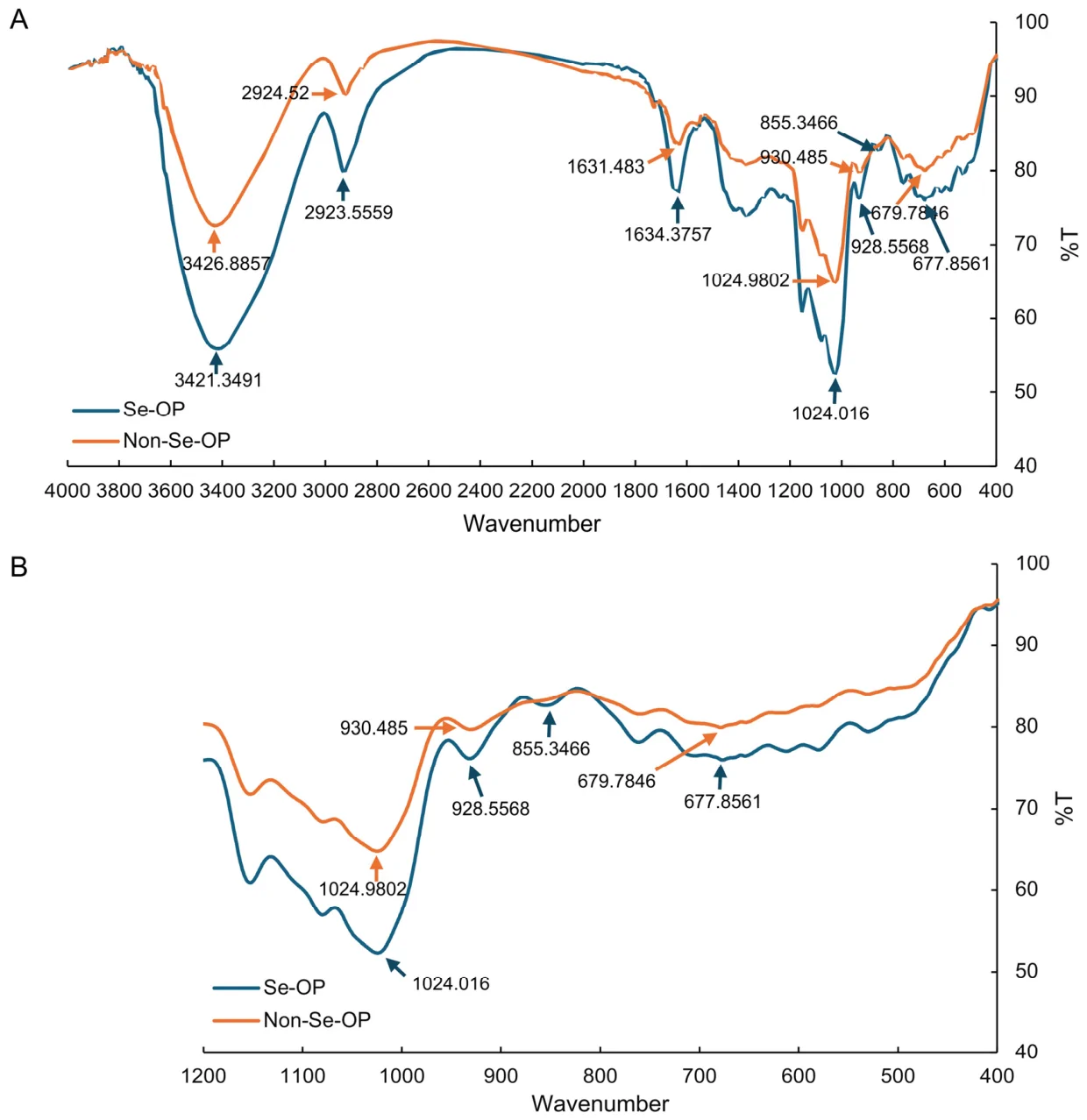
FTIR spectra of polysaccharides from non-selenium-enriched oats (OP0-3) and selenium-enriched oats (OP150-3): (**A**) full spectral range and (**B**) expanded fingerprint region (1200–400 cm^−1^).

The purified polysaccharides obtained from the non-selenium-associated polysaccharide fraction (OP0-3) and selenium-associated polysaccharide fraction (OP150-3) were characterized by Fourier transform infrared (FTIR) spectroscopy, as shown in Figure 7. The FTIR spectra of both samples exhibited similar overall spectral profiles and characteristic absorption bands, suggesting that the major structural features of the polysaccharides were largely preserved following selenium enrichment. A broad absorption band was observed at approximately 3426 and 3421 cm^−1^ for OP0-3 and OP150-3, respectively, and was assigned to the stretching vibration of hydroxyl (O–H) groups, while the bands at approximately 2924 and 2929 cm^−1^ corresponded to C–H stretching vibrations. The absorption bands at approximately 1631 and 1634 cm^−1^ were mainly attributed to the bending vibration of absorbed water associated with the polysaccharide matrix. In addition, the absorption band at approximately 1025 cm^−1^ was assigned to the stretching vibrations of C–O and C–O–C bonds, which are characteristic of polysaccharide structures [44,45].

Compared with OP0-3, OP150-3 exhibited a slight shift in the broad O–H stretching band and changes in several absorption features within the fingerprint region (1200–400 cm^−1^). In particular, the band near 930 cm^−1^ in OP0-3 was observed at approximately 932 cm^−1^ in OP150-3, while an additional absorption feature was observed at approximately 855 cm^−1^ in OP150-3. Changes were also observed in the lower-wavenumber region below 700 cm^−1^. These differences suggest that the chemical environment of some functional groups may have been altered following selenium enrichment. However, the relatively small shifts in band position should be interpreted cautiously because FTIR spectral variations may also result from differences in hydrogen bonding, moisture content, and sample preparation conditions [46].

The absorption features at 930 and 855 cm^−1^ following selenium enrichment provide supporting evidence for the formation of Se-containing linkages, such as O–Se–O, C–O–Se, and O–Se–C, which may be consistent with selenium modification of the polysaccharide [46]. The molecular confirmation cannot be established clearly from FTIR spectroscopy alone because the fingerprint region of polysaccharides contains multiple overlapping vibrations. Instead, the observed spectral differences may reflect changes in the chemical environment or interactions of oxygen-containing functional groups within the polysaccharide matrix following selenium enrichment. When considered together with the ICP-MS results confirming the presence of selenium in OP150-3, the FTIR data provide supporting evidence of changes in the chemical environment of the selenium-associated polysaccharide fraction following selenium enrichment. Nevertheless, FTIR spectroscopy alone cannot confirm covalent selenium–polysaccharide bonding or determine the specific binding site and bonding mode of selenium. Further characterization using high-performance size-exclusion chromatography coupled with multi-angle light scattering (HPSEC-MALLS) for molecular weight distribution, HPLC or GC-MS for monosaccharide composition, and NMR spectroscopy for structural elucidation would provide more comprehensive information regarding the molecular characteristics and specific mode of selenium association with the polysaccharide fraction.

## 4. Conclusions

In conclusion, oats exhibited a strong hyper-accumulation capacity for selenium, germinating successfully in sodium selenite concentrations up to 150 ppm without apparent cytotoxicity. Ultrasonic-assisted extraction under optimized conditions effectively enhanced extraction yield, while selenium fortification significantly elevated b-glucan content. Structural characterization showed that selenium enrichment might preserve the core polysaccharide backbone, inducing only subtle vibrational shifts indicative of selenium interaction with the polymer matrix. Moreover, the chromatographic fractionation isolated a key selenium-associated polysaccharide (OP150-3) characterized by high selenium integration and superior antioxidant capacity relative to its non-selenium-enriched counterpart, without cytotoxicity observed in cell studies. Overall, these findings suggest that selenium-enriched oats are a potential source of bioactive polysaccharides for functional food and nutraceutical applications, although further in-depth cytotoxicity studies and evaluation of health-related functional properties are warranted before those applications can be fully established.

## Figures and Tables

**Table 1:** Experimental design and results for analysis using Response Surface Methodology.

Run	Power (Watt)	Extraction Time (min)	Solid-to-Liquid (*w*/*v*%)	Crude Polysaccharide Extraction Yield (%)	Total Polysaccharide Content (mg/g Extract)	Total Selenium Content (mg Se/g Extract)
1	375	20	7.5	11.14 ± 0.19	728.90 ± 0.01	5.90 ± 0.87
2	375	20	7.5	12.35 ± 0.55	706.42 ± 0.01	4.72 ± 0.55
3	525	20	10	8.66 ± 0.21	697.16 ± 0.01	2.28 ± 0.06
4	375	10	5	5.81 ± 0.20	770.08 ± 0.02	3.24 ± 0.76
5	225	20	10	4.17 ± 0.18	756.06 ± 0.04	3.20 ± 0.43
6	525	10	7.5	6.29 ± 0.58	795.66 ± 0.11	3.08 ± 0.03
7	375	30	5	6.78 ± 0.74	953.61 ± 0.09	4.47 ± 0.25
8	225	10	7.5	5.70 ± 0.61	922.01 ± 0.02	2.68 ± 0.38
9	525	20	5	4.78 ± 0.20	976.39 ± 0.13	2.58 ± 0.22
10	225	30	7.5	7.51 ± 0.51	829.63 ± 0.06	2.81 ± 0.21
11	375	20	7.5	11.27 ± 0.01	812.95 ± 0.32	4.22 ± 0.04
12	225	20	5	7.34 ± 1.39	804.44 ± 0.21	3.56 ± 0.18
13	525	30	7.5	10.23 ± 0.13	959.53 ± 0.09	5.35 ± 0.03
14	375	10	10	5.64 ± 0.13	654.36 ± 0.06	2.39 ± 0.08
15	375	30	10	6.04 ± 0.25	744.68 ± 0.03	2.73 ± 0.21

**Table 2:** Summary of RSM model performance and validation results.

Parameters	Model (*p*-Value)	Lack-of-Fit	R^2^	Adjust R^2^	Predicted	Experimental	Relative Error (%)
**Total polysaccharide content**	0.0342	0.6335	0.9119	0.7534	980.29	939.45	4.35
**Crude polysaccharide extraction yield (%)**	0.0060	0.3220	0.9581	0.8826	9.82	9.96	1.41

**Table 3:** Selenium and β-glucan contents of DEAE-52 fractions obtained from selenium-treated (Se-OP) and control oats (Non-Se-OP).

Fractions	Selenium Content (mg Se/g Polysaccharide)		β-Glucan (g/100 g Dry Weight)	
	Se-OP	Non-Se-OP	Se-OP	Non-Se-OP
OP-1	ND	ND	4.56 ± 0.01 ^c,^*	2.79 ± 0.01 ^b,^*
OP-2	ND	ND	4.61 ± 0.01 ^a,^*	2.88 ± 0.01 ^b,^*
OP-3	0.12 ± 0.01 ^a^	ND	4.00 ± 0.01 ^d,^*	3.86 ± 0.01 ^a,^*
OP-4	0.02 ± 0.01 ^b^	ND	3.83 ± 0.01 ^e^	3.02 ± 0.01 ^c^
OP-5	ND	ND	2.97 ± 0.01 ^f,^*	2.85 ± 0.01 ^d,^*

Note: ND = not detected (below the detection limit). Values are expressed as mean ± SD (*n* = 3). Different lowercase letters (a–f) indicate significant differences among fractions within the same treatment (*p* < 0.05). * indicates a significant difference between Se-OP and Non-Se-OP within the same fraction (Independent-samples *t*-test, *p* < 0.05).

## Data Availability

The original contributions presented in this study are included in the article/Supplementary Material. Further inquiries can be directed to the corresponding author.

## Supplementary Materials

The following supporting information can be downloaded at: https://www.mdpi.com/article/10.3390/foods15183241/s1, Figure S1: Effects of selenium concentration on the changes in oat seeds over a 4-day period; Table S1: Analysis of variance (ANOVA) for the quadratic models.

## Abbreviations

The following abbreviations are used in this manuscript:

UAE	Ultrasound-Assisted Extraction
FTIR	Fourier Transform Infrared Spectroscopy
ICP	Inductively Coupled Plasma
Se-OP	Selenium-enriched oat polysaccharide
