# Supplementary material for: Optimization of Selenium Fortification and Ultrasound-Assisted Extraction of Bioactive Selenium-Polysaccharide Fractions from Germinated Oats (Avena sativa)
Source: Foods. 2026 Sep 14;15(18):3241. doi: 10.3390/foods15183241 (PMC13606241; doi:10.3390/foods15183241)
Supplement: Supplementary file 1 [file foods-15-03241-s001.zip › foods-4524247-supplementary.pdf]

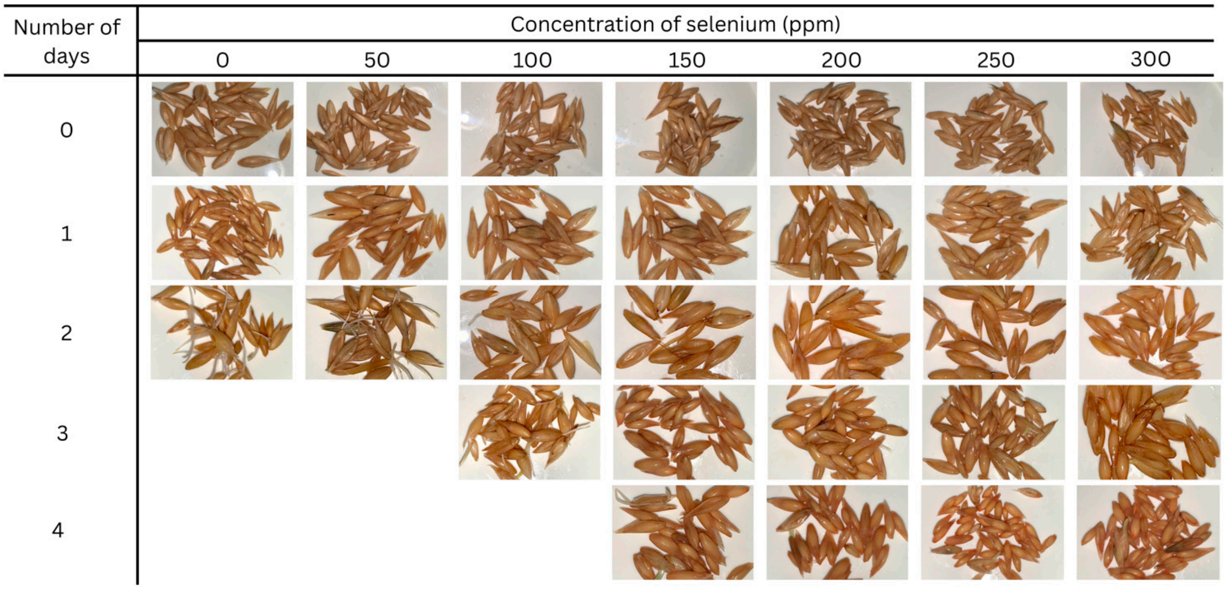

Figure S1. Effects of selenium concentration on the changes of oat seeds over a 4-day period.

Supplementary Table S1. Analysis of variance (ANOVA) for the quadratic models

Response	Source	Sum of Squares	df	Mean Square	F-value	p-value
**Crude polysaccharide yield (%)**	**Model**	88.07	9	9.79	12.70	0.0060
	A	3.45	1	3.45	4.48	0.0879
	B	6.35	1	6.35	8.24	0.0349
	C	4.790E-003	1	4.790E-003	6.214E-003	0.9402
	A^2	14.65	1	14.65	19.01	0.0073
	B^2	17.65	1	17.65	22.40	0.0052
	C^2	17.26	1	17.26	54.01	0.0007
	AB	41.63	1	41.63	1.47	0.2790
	AC	1.14	1	1.14	16.12	0.0102
	BC	0.080	1	0.080	0.10	0.7603
	Residual	3.58	5	0.77		
	Lack of Fit	2.97	3	0.99		
	Pure Error	0.88	2	0.44	2.25	0.3220
	Cor Total	91.93	14			
**Total polysaccharide**	**Model**	0.13	9	0.015	5.75	0.0342
	A	1.700E-003	1	1.700E-003	0.66	0.4543
	B	0.015	1	0.015	5.77	0.0615

**content -mg/g# extract,**	C	0.053	1	0.053	20.57	0.0062
	A^2	0.022	1	0.022	8.59	0.0326
	B^2	9.131E-003	1	9.131E-003	3.53	0.1190
	C^2	1.259E-003	1	1.259E-003	0.49	0.5163
	AB	0.016	1	0.016	6.35	0.0532
	AC	0.013	1	0.013	5.15	0.0724
	BC	2.172E-003	1	2.172E-003	0.84	0.4014
	Residual	0.013	5	2.585E-003		
	Lack of Fit	6.619E-003	3	2.206E-003	0.70	0.6335
	Pure Error	6.619E-003	2	3.153E-003		
	Cor Total	0.15	14			
	**Model**	12.77	9	1.42	1.43	0.3632
	A	0.14	1	0.14	0.14	0.7256
	B	1.96	1	1.96	1.97	0.2194
	C	1.31	1	1.31	1.32	0.3028
	A^2	2.88	1	2.88	2.90	0.1494
**Total selenium content -mg Se/g extract,**	B^2	1.26	1	1.26	1.27	0.3113
	C^2	4.96	1	4.96	4.99	0.0758
	AB	1.14	1	1.14	1.15	0.3326
	AC	9.515E-004	1	9.515E-004	9.571E-004	0.9765
	BC	0.20	1	0.20	0.20	0.6747
	Residual	4.97	5	0.99		
	Lack of Fit	3.48	3	1.16	1.55	0.4149
	Pure Error	1.49	2	0.75		
	Cor Total	17.74	14			
